# Long-Lived CD4^+^IFN-γ^+^ T Cells rather than Short-Lived CD4^+^IFN-γ^+^IL-10^+^ T Cells Initiate Rapid IL-10 Production To Suppress Anamnestic T Cell Responses during Secondary Malaria Infection

**DOI:** 10.4049/jimmunol.1600968

**Published:** 2016-09-14

**Authors:** Ana Villegas-Mendez, Colette A. Inkson, Tovah N. Shaw, Patrick Strangward, Kevin N. Couper

**Affiliations:** Faculty of Biology, Medicine and Health, University of Manchester, Manchester M13 9PT, United Kingdom

## Abstract

CD4^+^ T cells that produce IFN-γ are the source of host-protective IL-10 during primary infection with a number of different pathogens, including *Plasmodium* spp. The fate of these CD4^+^IFN-γ^+^IL-10^+^ T cells following clearance of primary infection and their subsequent influence on the course of repeated infections is, however, presently unknown. In this study, utilizing IFN-γ–yellow fluorescent protein (YFP) and IL-10–GFP dual reporter mice, we show that primary malaria infection–induced CD4^+^YFP^+^GFP^+^ T cells have limited memory potential, do not stably express IL-10, and are disproportionately lost from the Ag-experienced CD4^+^ T cell memory population during the maintenance phase postinfection. CD4^+^YFP^+^GFP^+^ T cells generally exhibited a short-lived effector rather than effector memory T cell phenotype postinfection and expressed high levels of PD-1, Lag-3, and TIGIT, indicative of cellular exhaustion. Consistently, the surviving CD4^+^YFP^+^GFP^+^ T cell–derived cells were unresponsive and failed to proliferate during the early phase of secondary infection. In contrast, CD4^+^YFP^+^GFP^−^ T cell–derived cells expanded rapidly and upregulated IL-10 expression during secondary infection. Correspondingly, CD4^+^ T cells were the major producers within an accelerated and amplified IL-10 response during the early stage of secondary malaria infection. Notably, IL-10 exerted quantitatively stronger regulatory effects on innate and CD4^+^ T cell responses during primary and secondary infections, respectively. The results in this study significantly improve our understanding of the durability of IL-10–producing CD4^+^ T cells postinfection and provide information on how IL-10 may contribute to optimized parasite control and prevention of immune-mediated pathology during repeated malaria infections.

## Introduction

The cytokine IL-10 plays a central role in determining the outcome of many different infections, including malaria (1, 2). In murine models of primary malaria infection, IL-10 is critical for repressing the development of immune-mediated pathology in tissues, including the liver, lung, and brain (3–7). In agreement, levels of IL-10 are frequently lower in individuals with severe *Plasmodium falciparum* infections compared with individuals with mild or asymptomatic infections (8, 9). Nevertheless, in both human and murine malaria infections, overproduction or mistimed production of IL-10 can also blunt protective immune responses during infection, resulting in high parasite burdens and morbidity (10, 11). Although the precise mechanisms of action of IL-10 during malaria infection remain to be defined, it has been shown to suppress the production of proinflammatory cytokines, including TNF, IFN-γ, and IL-12 (4, 6). In other models, IL-10 can directly suppress the inflammatory activity of multiple cell types within the innate and adaptive immune compartments, including macrophages, dendritic cells, T cells, and B cells (1, 2, 12).

CD4^+^ T cells, and in particular the Th1 subset, are the major source of IL-10 during both murine and human malaria infections (3, 5, 13, 14). As a consequence, IL-10–producing Th1 cells are nonredundantly required for attenuation of morbidity and immune-mediated pathology during primary murine malaria infection (3, 5). At present, however, the fate and the memory potential of these IL-10–producing Th1 cells following clearance of primary malaria infection remains unclear, both in mice and in humans. A number of the signals that instruct IL-10 expression by Th1 cells during primary malaria infection, including IL-27R and ICOS, play major roles in programming the development, maintenance, and function of memory T cell populations (15–18), implying that IL-10–producing Th1 cells may have a selective advantage in transitioning into long-lived memory cells. In apparent agreement, it has been reported that durable parasite-specific IL-10–, but not IFN-γ–, producing CD4^+^ T cell responses can be sustained in individuals many years after malaria infection (19). However, in contrast to the results reported by Wipasa et al. (19), long-lived IFN-γ–producing activated CD4^+^ T cells have been observed during malaria and multiple other infections (20–22). Moreover, it has recently been suggested that *P. falciparum*–induced IL-10 production is maintained only during the duration of active malaria infection (23), and that the frequencies of maintained IL-10–producing CD4^+^ T cells are inversely correlated with duration from previous exposure (14). These latter results are consistent with the divergent or linear limited potential models of memory T cell development where fully differentiated effector T cells—it is currently thought that IL-10 expression is induced in fully differentiated effector Th1 cells (24, 25)—have limited capacity to form memory T cells (26, 27). Thus, it remains to be definitively resolved, in any model, whether IL-10–producing Th1 cells have the capacity to become long-lived memory cells that subsequently regulate the nature and strength of anamnestic immune responses during secondary infections.

Of relevance, it has been hypothesized that infection-acquired protection against severe malaria disease (termed antidisease immunity) involves development of immune mechanisms that attenuate the strong inflammatory responses that contribute toward much of the morbidity and mortality of initial malaria infections (9, 28). At present, the molecular and cellular basis of protective anamnestic immune responses to malaria are unclear (9, 28); however, given the importance of IL-10 in regulating inflammatory responses during malaria infection (1–9), it is foreseeable that IL-10 may play a major role in acquired (antidisease) protection against severe malarial disease. Nevertheless, whether primary parasite exposure leads to qualitative or quantitative reprogramming of the dynamics of the IL-10 response during subsequent malaria infections, and whether IL-10 exerts stronger or dichotomous activity during secondary versus primary malaria infections, has yet to be definitively addressed.

In this study, utilizing a novel tractable IFN-γ and IL-10 dual reporter system, we have shown that IL-10–producing Ag-experienced CD4^+^ T cells generated during the early phases of primary malaria infection have limited ability to populate the memory CD4^+^ T cell compartment that functions during subsequent parasite infections. However, CD4^+^IFN-γ–yellow fluorescent protein (YFP)^+^IL-10–GFP^−^ memory T cells rapidly express IL-10 during parasite re-exposure, which leads to enhanced IL-10 production during secondary malaria infection compared with primary infection. We have addressed the impact of elevated IL-10 production during secondary infection in shaping anamnestic immune responses and demonstrate that IL-10 may play an important role in regulating reactivation of memory CD4^+^ T cells. Our results significantly increase our understanding of the dynamics and maintenance of IL-10 production by CD4^+^ T cells postinfection and reveal the functions of IL-10 during secondary parasite infections.

## Materials and Methods

### Ethics

All animal work was approved following local ethical review by the University of Manchester Animal Procedures and Ethics Committee and was performed in strict accordance with the U.K. Home Office Animals (Scientific Procedures) Act 1986 (project license 70/7293).

### Mice and parasites

IFN-γ YFP reporter mice (29), IL-10 GFP reporter mice (30), and C57BL/6 CD45.1^+^ (Pep3) mice were bred and maintained at the University of Manchester in individual ventilated cages. Heterozygous IFN-γ reporters and homozygous IL-10 reporter mice were intercrossed to generate dual reporter mice. F_1_ offspring were used in experiments after genotyping for YFP expression by flow cytometry. Sex-matched 6- to 10-wk-old mice were used in separate experiments.

Cryopreserved *Plasmodium yoelii* NL parasites were thawed and passaged through C57BL/6 mice. Experimental mice were subsequently infected with 1 × 10^4^ parasitized RBCs (pRBCs) via i.v. injection in the tail vein. The course of infection was monitored by microscopic examination of peripheral parasite levels in Giemsa-stained thin blood smears and by assessing weight loss (calculated relative to uninfected starting weight). To terminate primary infection at a defined time point, mice were treated with pyrimethamine in drinking water from day 9 to day 19 of infection. Drugs were also administered to age-matched uninfected mice used as uninfected or primary infection controls. In some experiments, previously infected mice and age-matched controls were infected with 1 × 10^4^ pRBCs on day 60 after primary infection (secondary infection). In some instances, experimental mice were injected i.p. with 250 μg anti–IL-10R (Bio X Cell, West Lebanon, NH) on days −1, +1, +3, and +5 of primary and secondary infection.

### Flow cytometry

Spleens were obtained and animals were subsequently perfused by intracardial injection of 10 ml of PBS before other organs were removed. Single-cell suspensions from spleen and liver were prepared by homogenization through a 70-μm cell strainer (BD Biosciences). RBCs were lysed (RBC lysing buffer; BD Biosciences) and samples were washed and resuspended in FACS buffer (HBSS with 2% FCS). Blood, obtained by cardiac puncture prior to whole-body perfusion, was treated with RBC lysis buffer and resuspended in FACS buffer. Lungs were dissected into chunks and incubated in HBSS containing 2% FCS with collagenase (final concentration 1 mg/ml; Sigma-Aldrich) for 45 min on a tube roller at room temperature. The resulting suspension was filtered through a 70-μm cell strainer and RBCs were lysed. Samples were refiltered through a 70-μm cell strainer, washed, and resuspended in FACS buffer. Bone marrow (BM) cells were obtained from the femur by flushing with FACS buffer. Live/dead cell differentiation and absolute cell numbers were calculated by trypan blue exclusion (Sigma-Aldrich) using a C-Chip (NanoEnTek, Pleasanton, CA).

Following staining with Live/Dead fixable blue dead cell stain for UV (Life Technologies), CD4^+^ T cell phenotyping was performed by surface staining with anti-mouse Abs against CD4 (GK1.5), CD45.1 (A20), CD45.2 (104), CD44 (IM7), CD62L (MEL-14), CD49d (R1-2), CD11a (M17/4), CD127 (A7R34),KLRG1 (2F1), PD-1 (RMP1-30), CD25 (PC61), LAG-3 (C9B7W), CD69 (H1.2F3), ICOS (C398.4A), TIGIT (GIGD7), and CD103 (2E7). Different lymphoid populations were assessed by surface staining with CD3 (17A2), CD40 (1C10), MHC class II (M5/114.15.2), CD8a (53-6.7), CD11c (N418), CD68 (Fa-11), CD49b (DX5), F4-80 (BM8), Ly6C (HK1.4), Ly6G (RB6.8C5), CD19 (6D5), and CD11b (M1/70). All staining cocktails contained a mouse Fc receptor block (clone Fc-G2a).

All Abs were purchased from eBioscience or BioLegend. Fluorescence minus one controls were used to validate flow cytometric results. All flow cytometry acquisition was performed using an LSR II (BD Systems, U.K.) under the same application settings, with a configuration including 510/20 nm bandpass and 500 nm longpass (for GFP signal) and 550/30 nm bandpass and 525 nm longpass (for YFP signal) filters. All FACS analyses were performed using FlowJo software (Tree Star, Ashland, OR).

### Adoptive transfer experiments

Splenic CD4^+^ T cells from malaria-infected (day 7) dual reporter mice were purified by magnetic positive selection using anti-CD4 MicroBeads (Miltenyi Biotec) according to the manufacturer’s guidelines. CD4^+^ T cells were then stained with anti-mouse CD49d and anti-mouse CD11a (as described above). Total CD4^+^CD49d^+^CD11a^+^ T cells and YFP^+^GFP^−^ and YFP^+^GFP^+^ subsets were isolated by flow cytometric cell sorting (BD FACSAria). The different populations of purified cells were adoptively transferred, separately, by i.v. injection into malaria-infected (day 7) congenic CD45.1^+^ mice, which were subsequently drug cured as described above. In some experiments recipient mice were reinfected with 1 × 10^4^ pRBCs on day 60 after primary infection.

### In vitro T cell restimulation assays

CD4^+^CD49d^+^CD11a^+^ T cells were sort purified, as described above, from the spleens of day 7 and day 60 infected Pep3 mice. As a control, total CD4^+^ T cells were purified from spleens of naive mice by magnetic separation. Cells were cultured at 2 × 10^5^ cell density in 96-well plates and stimulated for either 4 h with 200 ng/ml PMA (Sigma-Aldrich) and 1 μg/ml ionomycin (Sigma-Aldrich) or 24 and 72 h with 2 μg/ml anti-CD3 (BD Biosciences) and 2 μg/ml anti-CD28 (eBioscience). Cell supernatants were removed and stored at −80°C. The concentrations of IL-2, IFN-γ, and IL-10 in supernatants were measured by a cytometric bead array mouse Th1/Th2/Th17 cytokine kit (BD Biosciences), following the manufacturer’s instructions.

### Quantification of plasma cytokine levels

The concentrations of multiple innate and adaptive-derived cytokines and chemokines in plasma were measured by ProcartaPlex 26-plex mouse cytokine and chemokine panel (eBioscience) using a Luminex 100/200 system, following the manufacturer’s instructions.

### Statistical analysis

For two group comparisons, statistical significance was determined using a *t* test. For three or more group comparisons, statistical significance was determined using a one-way or two-way ANOVA, with a Bonferroni post hoc analysis. Results were considered as significantly different when *p* < 0.05

## Results

### CD4^+^YFP^+^GFP^+^ T cells are disproportionately lost within the Ag-experienced CD4^+^ T cell compartment after clearance of primary malaria infection

To date, the fate of CD4^+^IFN-γ^+^IL-10^+^ T cells postinfection and their capacity to enter into the long-lived (memory) CD4^+^ T cell pool has not been assessed in detail in any model. Thus, to definitively investigate whether a long-lived CD4^+^IFN-γ^+^IL-10^+^ T cell population is maintained after malaria infection, we generated dual IFN-γ–YFP and IL-10–GFP reporter mice and determined the kinetics of the Ag-experienced CD4^+^YFP^+^GFP^+^ T cell response during and after primary malaria infection. We assessed the expansion and contraction of the CD4^+^YFP^+^GFP^+^ T cell response in the spleen, BM, and selected nonlymphoid organs to identify whether particular tissue sites provided niche environments for the preferential survival or maintenance of CD4^+^YFP^+^GFP^+^ T cells. Because tetramers do not exist for tracking parasite-specific CD4^+^ T cell responses during *P. yoelii* NL infection, we used CD11a and CD49d as proxy markers for activated Ag-experienced cells, which include malaria-specific effector and memory CD4^+^ T cell populations (31, 32). Of note, IL-10 is only expressed by the CD11a^+^CD49d^+^ subset during malaria infection (32).

**FIGURE 1.**
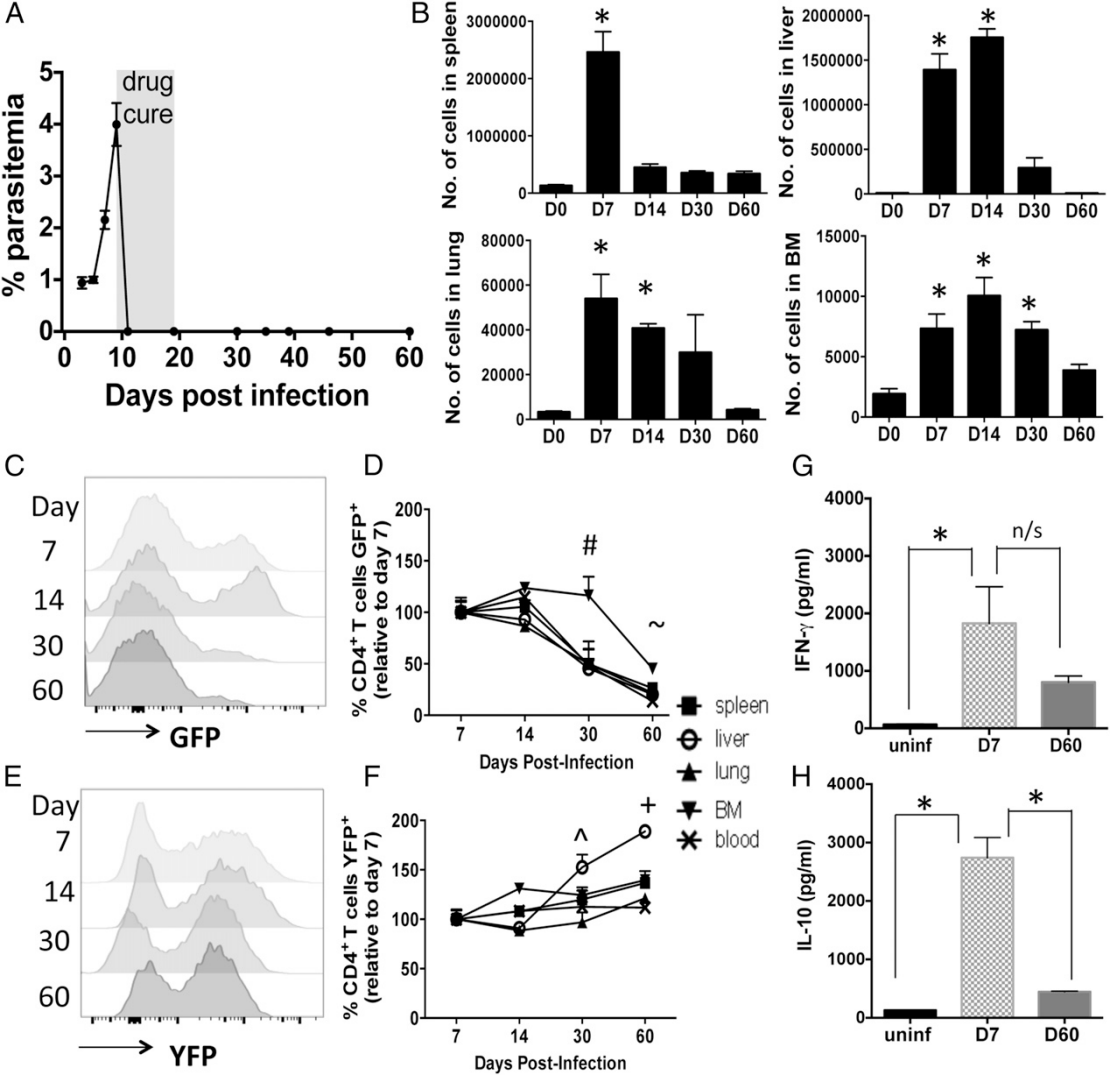
The contraction kinetics of the Ag-experienced CD4^+^YFP^+^GFP^+^ T cell response after primary malaria infection. IFN-γ–YFP and IL-10–GFP dual reporter mice were infected (i.v.) with 1 × 10^4^ *P. yoelii* NL pRBCs. Mice were treated with pyrimethamine from day 9 of infection for 10 d. (**A**) Peripheral parasite burdens before and after drug treatment. (**B**) The numbers of Ag-experienced (CD11a^+^CD49d^+^) CD4^+^YFP^+^GFP^+^ T cell populations in various organs before and after drug treatment. (**C**–**F**) Representative histograms (spleen) and calculated relative maintenance (to day 7 postinfection levels) of (C and D) CD4^+^YFP^+^GFP^+^ and (E and F) CD4^+^YFP^+^ cells within the Ag-experienced CD4^+^ T cell population after malaria infection. (**G** and **H**) Splenic CD4^+^CD11a^+^CD49d^+^ cells were sort purified from mice on day 7 and day 60 postinfection and total CD4^+^ T cells were purified from uninfected mice (uninf). Cells were stimulated for 4 h with PMA and ionomycin and supernatant was assayed for (G) IFN-γ production and (H) IL-10 production. The results are the mean ± SEM of the group from two to three independent experiments (each experiment with three to five mice). (B) *p < 0.05 between numbers of CD4^+^YFP^+^GFP^+^ T cells on specified day versus numbers on day 0. (D) ^#^*p* < 0.05 between all tissues except BM (day 30) versus respective day 7 level. ^∼^*p* < 0.05 between all tissues (day 60) versus respective day 7 level. (F) ^*p* < 0.05 between liver (day 30) versus respective day 7 level. ^+^*p* < 0.05 between spleen, liver, and BM (day 60) versus respective day 7 level. (G and H) *p < 0.05 between annotated groups. Significance tested using (B, G, and H) one-way ANOVA test with a Bonferroni post hoc analysis and (D and F) two-way ANOVA with a Bonferoni post hoc analysis.

As previously described (32), the numbers of IFN-γ–YFP and IL-10–GFP dual-expressing CD4^+^CD11a^+^CD49d^+^ T cells increased significantly in all examined organs by day 7 of infection, before antimalarial drug administration (Fig. 1A, 1B). Following antimalarial drug administration, used to terminate infection at a defined time point and to replicate chemotherapy of human malaria infection, the numbers of Ag-experienced CD4^+^YFP^+^GFP^+^ T cells declined heterogeneously in the examined tissues, with the contraction being extremely fast in the spleen and slowest in the BM (Fig. 1B). By day 60 postinfection, the numbers of CD4^+^YFP^+^GFP^+^ T cells had returned to approximately baseline levels in all examined organs, indicating that Ag-experienced CD4^+^YFP^+^GFP^+^ T cells were not maintained long-term in any anatomical compartment after malaria infection (Fig. 1B).

Consistent with the above data, IL-10–GFP^+^ T cells were disproportionately lost within the Ag-experienced CD4^+^ T cell population after malaria infection during the memory maintenance phase (which we define as the interval between clearance of infection and the time of reinfection) (Fig. 1C, 1D). By day 60 postinfection the frequencies of CD4^+^YFP^+^GFP^+^ T cells within the Ag-experienced CD4^+^ T cell response were ∼20–25% of those observed on day 7 (Fig. 1C, 1D). This is in contrast to the frequencies of YFP^+^GFP^−^ cells within the Ag-experienced CD4^+^ T cell pool, which was stably maintained (or increased) in all examined tissues between days 14 and 60 postinfection (Fig. 1E, 1F). In agreement with the reporter data, Ag-experienced splenic CD4^+^ T cells obtained on day 7 of infection produced high levels of IFN-γ and IL-10 protein (Fig. 1G, 1H). Moreover, whereas Ag-experienced CD4^+^ T cells obtained on day 60 of infection produced only marginally less IFN-γ, they produced significantly less IL-10 than did counterpart cells obtained from day 7 of infection (Fig. 1G, 1H). Collectively, these data show that CD4^+^YFP^+^GFP^+^ T cells are less able to populate the long-lived Ag-experienced CD4^+^ T cell compartment than are other CD4^+^ T cell subsets after malaria infection. Furthermore, IL-10 production is extinguished rapidly within the Ag-experienced CD4^+^ T cell compartment postinfection.

### CD4^+^YFP^+^GFP^+^ T cells exhibit a short-lived and exhausted effector phenotype that restricts their entry into the long-lived Ag-experienced CD4^+^ T cell pool

**FIGURE 2.**
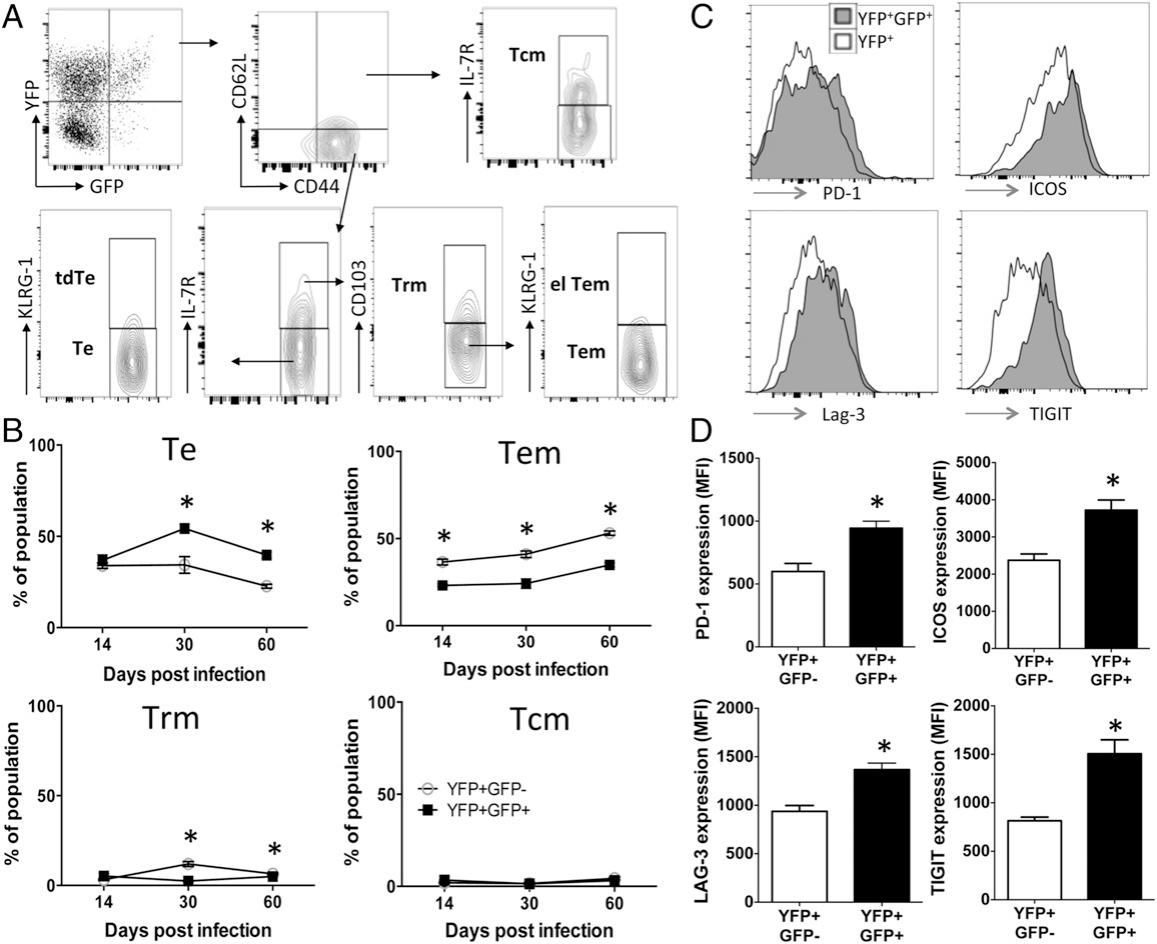
Ag-experienced CD4^+^YFP^+^GFP^+^ T cells are short-lived effector cells and express markers of cellular exhaustion after malaria infection. IFN-γ–YFP and IL-10–GFP dual reporter mice were infected (i.v.) with 1 × 10^4^ *P. yoelii* NL pRBCs. Mice were treated with pyrimethamine from day 9 of infection for 10 d. (**A**) Representative dot plots demonstrating the gating strategy and characterization of effector and memory CD4^+^ T cell subsets within the splenic parasite-specific CD4^+^YFP^+^GFP^+^ T cell population on day 14 postinfection. (**B**) Breakdown of the splenic Ag-experienced CD4^+^YFP^+^GFP^+^ T cell and CD4^+^YFP^+^GFP^−^ T cell populations into effector and memory subsets postinfection. (**C** and **D**) Representative histograms showing the expression of cell surface markers on splenic CD4^+^YFP^+^GFP^+^ and CD4^+^YFP^+^GFP^−^ T cells on day 60 postinfection. (D) Calculated mean fluorescence intensity (MFI) of expression of markers by CD4^+^YFP^+^GFP^+^ and CD4^+^YFP^+^GFP^−^ T cells on day 60 postinfection. The results are the mean ± SEM of the group with three to five mice per group, and are representative of three independent experiments. (B and D) *p < 0.05. Significance was tested using an unpaired two-tailed *t* test. el Tem, effector-like effector memory T cell; Tcm, central memory T cell; tdTe, terminally differentiated effector T cell; Te, effector T cell; Tem, effector memory T cell; Trm, resident memory T cell.

The disproportionate loss of the CD4^+^YFP^+^GFP^+^ T cells within the Ag-experienced CD4^+^ T cell pool after clearance of malaria infection indicated that the CD4^+^YFP^+^GFP^+^ T cells may exhibit specific cellular characteristics that restricted their maintenance. To examine this, CD4^+^YFP^+^GFP^+^ T cells and, for comparison, CD4^+^YFP^+^GFP^−^ T cells that survived postinfection were first subcharacterized into various populations of effector and memory T cell subsets (Fig. 2A). As anticipated, high frequencies of both CD4^+^YFP^+^GFP^+^ T cells and CD4^+^YFP^+^GFP^−^ T cell subsets exhibited an effector T cell phenotype (CD44^+^CD62L^low^, IL-7R^−^, KLRG-1^−^) on day 14 of infection; however, at later time points after clearance of infection (days 30 and 60) there was a significant divergence in phenotypes of CD4^+^YFP^+^GFP^+^ T cells and CD4^+^YFP^+^GFP^−^ T cells, with significantly higher frequencies of CD4^+^YFP^+^GFP^+^ T cells exhibiting an effector T cell profile compared with CD4^+^YFP^+^GFP^−^ T cells (Fig. 2B). Correspondingly, a significantly higher frequency of CD4^+^YFP^+^GFP^−^ T cells exhibited an effector memory T cell signature (CD44^+^CD62L^low^, IL-7R^+^, CD103^−^KLRG-1^−^) after clearance of infection compared with CD4^+^YFP^+^GFP^+^ T cells (Fig. 2B), and the proportion of CD4^+^YFP^+^GFP^−^ T cells exhibiting an effector memory T cell phenotype increased with time during the memory maintenance phase postinfection (Fig. 2B). Thus, the loss of CD4^+^YFP^+^GFP^+^ T cells after malaria infection (Fig. 1) appears to be related to their skewed differentiation as short-lived effector T cells and their inability to acquire durable effector memory T cell characteristics. Of note, very few CD4^+^YFP^+^GFP^+^ T cells and CD4^+^YFP^+^GFP^−^ T cells displayed phenotypic characteristics of resident memory (CD44^+^CD62L^low^, IL-7R^+^, CD103^+^), central memory (CD44^+^CD62L^high^, IL-7R^+^), terminally differentiated effector (CD44^+^CD62L^low^, IL-7R^−^, KLRG-1^+^), or effector-like effector memory T cells (CD44^+^CD62L^low^, IL-7R^+^, CD103^−^, KLRG-1^+^) on any day after clearance of malaria infection (Fig. 2A, 2B and results not shown).

It is clear that in addition to differentiation status, the activation profile of T cells may determine their ability to populate the long-lived memory T cell compartment postinfection (22, 33). CD4^+^YFP^+^GFP^+^ T cells expressed significantly higher levels of markers associated with cellular activation and exhaustion/regulation, including PD-1, ICOS, Lag3, and TIGIT on days 30 and 60 compared with CD4^+^YFP^+^GFP^−^ T cells (Fig. 2C and results not shown). Thus, these data suggest that the activation profile of CD4^+^YFP^+^GFP^+^ T cells, as well as their differentiation status, may restrict their maintenance after malaria infection.

### Malaria infection–induced CD4^+^YFP^+^GFP^+^ T cells are short-lived and are not stably programmed for IL-10 expression

**FIGURE 3.**
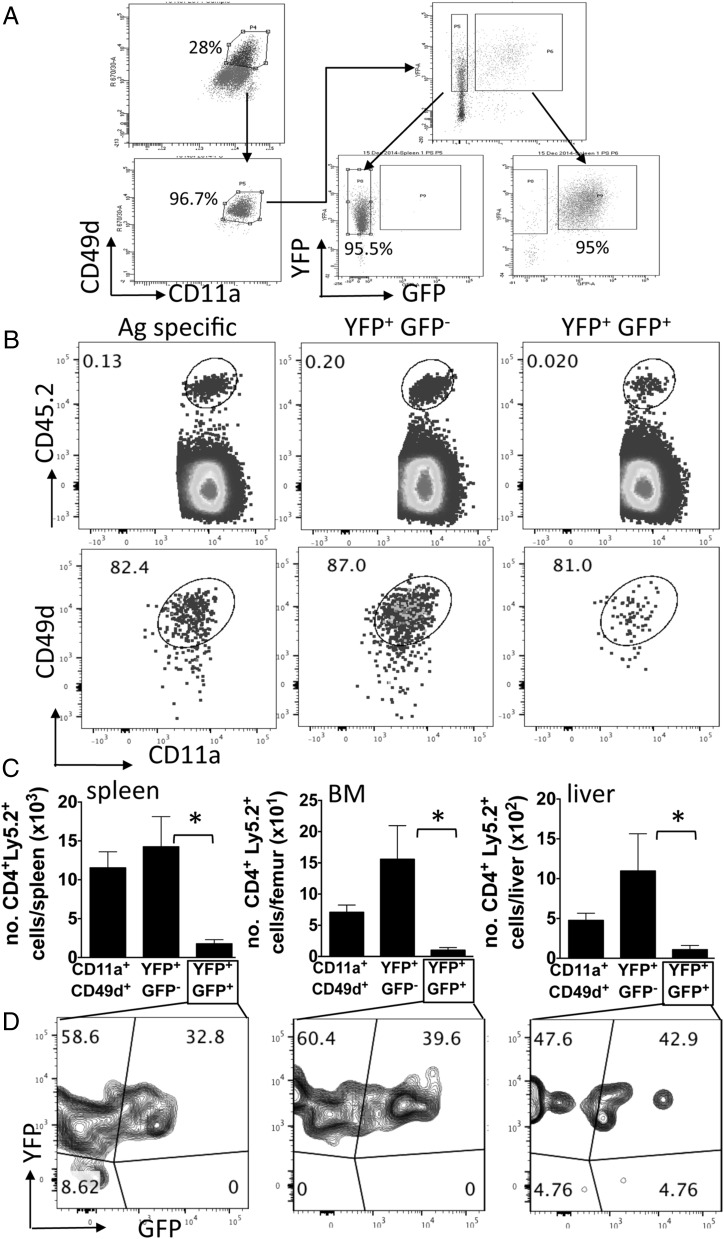
Ag-experienced CD4^+^YFP^+^GFP^+^ T cells have limited memory cell potential and are not programmed for stable IL-10 expression after malaria infection. Splenic Ag-experienced (CD11a^+^CD49d^+^) and Ag-experienced YFP^+^GFP^+/−^ subsets were sort purified from *P.yoelii* NL–infected (day 7: 1 × 10^4^ pRBCs [i.v.]) dual reporter mice (CD45.2^+^) and were adoptively transferred separately into infected (day 7 postinfection) CD45.1^+^ mice. Recipient mice were treated with pyrimethamine from day 9 of infection for 10 d. (**A**) Representative dot plots showing the gating and purity of the three Ag-experienced CD4^+^ T cell populations. (**B**) Representative dot plots showing the identification of the three adoptively transferred populations and their expression levels of CD11a CD49d in spleens of recipient mice on day 60 postinfection (53 d after transfer). (**C**) Numbers of the three populations of transferred cells maintained in spleen, BM, and liver of recipient mice on day 60 postinfection. (**D**) Representative dot plots showing the expression of IFN-γ–YFP and IL-10–GFP in adoptively transferred CD4^+^YFP^+^GFP^+^ T cell–derived cells in the spleen, BM, and liver of recipient mice on day 60 postinfection. The results are the mean ± SEM of the group with three to five mice per group and are representative of three independent experiments. *p < 0.05. Significance was tested using a one-way ANOVA test with a Bonferroni post hoc analysis.

The failure of the CD4^+^YFP^+^GFP^+^ T cells to express characteristics of memory T cells after malaria infection indicated that the CD4^+^YFP^+^GFP^+^ T cells were lost from the Ag-experienced CD4^+^ T cell population after infection because they were short-lived and rapidly died postinfection. To directly investigate this, total splenic Ag-experienced CD4^+^ T cells (CD11a^+^CD49d^+^) and Ag-experienced YFP^+^GFP^−^ and YFP^+^GFP^+^ subsets were purified from dual reporter mice on day 7 of infection (Fig. 3A), and equal numbers were adoptively transferred, separately, into infected CD45.1 congenic mice (day 7 of infection). Thus, donor T cell populations were transferred into the same physiological environment (i.e., Ag load and cytokine milieu) from which they were obtained, ensuring that the memory capacity of the cells could be appropriately defined. Utilizing the established drug cure model, recipient mice were treated with antimalarial drugs on day 9 to clear infection, and the maintenance of the donor populations was examined on day 60 postinfection. All three adoptively transferred populations were identified in the spleen on day 60 postinfection and they remained CD11a^+^ and CD49d^+^, verifying the validity of the surrogate marker system (Fig. 3B). Significantly fewer CD4^+^YFP^+^GFP^+^ T cells were, however, recovered in the spleen than CD4^+^YFP^+^GFP^−^ T cells, and a lower trend in recovery of CD4^+^YFP^+^GFP^+^ T cells was observed compared with total Ag-experienced CD4^+^ T cells (Fig. 3C). The reduced maintenance of transferred CD4^+^YFP^+^GFP^+^ T cells in the spleen compared with the CD4^+^YFP^+^GFP^−^ T cell population was not because they preferentially accumulated within other tissues, as fewer CD4^+^YFP^+^GFP^+^ T cells were recovered in the BM and liver than CD4^+^YFP^+^GFP^−^ or total Ag-experienced T cell populations (Fig. 3C).

Notably, in addition to their apparent short life span, a high proportion of the transferred donor CD4^+^YFP^+^GFP^+^ T cells had extinguished GFP expression, to a similar degree in all the tissues analyzed, by day 60 postinfection (Fig. 3D). Consequently, as the Vert-X mice are transcriptional reporters (30), these data imply that uniform and stable transcription of IL-10 mRNA does not occur in malaria-specific activated CD4^+^YFP^+^GFP^+^ T cells during and/or after malaria infection, suggesting that the IL-10 gene is not epigenetically imprinted for poised production in these cells. Alternatively, the IL-10 gene may be epigenetically silenced in the CD4^+^YFP^+^GFP^+^ T cells postinfection, actively inhibiting gene expression. In contrast, consistent with data in Fig. 1, donor CD4^+^YFP^+^GFP^+^ T cells maintained IFN-γ–YFP expression (Fig. 3D). As the YETI mice are also transcriptional reporters (29), this may suggest that the IFN-γ gene is epigenetically modified in IFN-γ–producing (IL-10–GFP^+/−^) CD4^+^ T cells, leading to poised expression of the cytokine mRNA in Th1-derived memory cells after malaria infection, as has been shown in NK cells and CD8^+^ T cells in other models (29, 34). This is consistent with the ability of Ag-experienced CD4^+^ T cells to maintain IFN-γ protein production postinfection (Fig. 1G).

Collectively, these data show that not only are CD4^+^YFP^+^GFP^+^ T cells inherently short-lived compared with other Ag-experienced CD4^+^ T cell subsets, but they fail to stably express IL-10 postinfection.

### CD4^+^YFP^+^GFP^+^ T cell–derived and CD4^+^YFP^+^GFP^−^ T cell–derived memory cells respond differently during rechallenge infections

**FIGURE 4.**
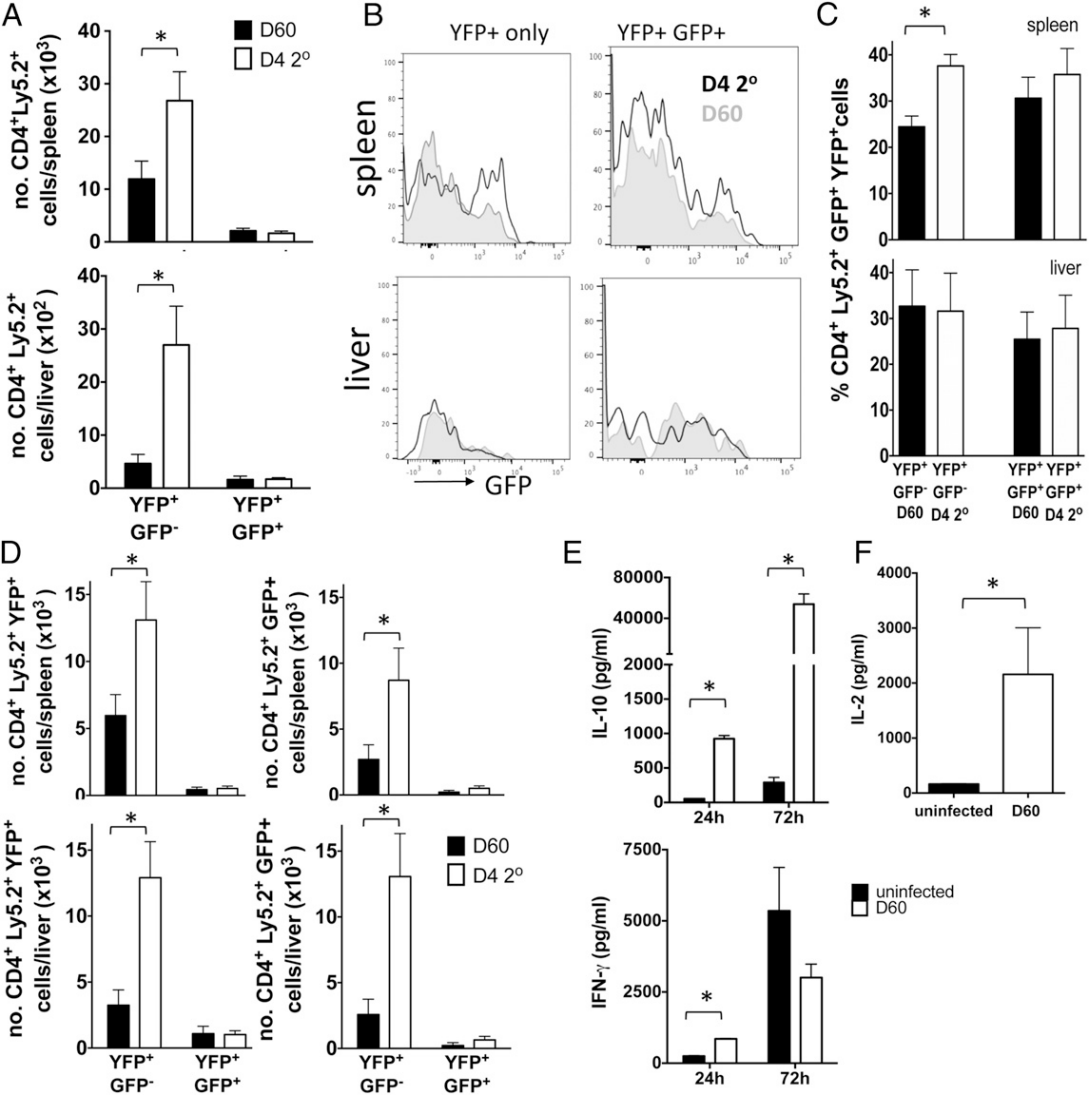
Ag-experienced CD4^+^YFP^+^YFP^−^ T cell–derived memory cells, but not CD4^+^YFP^+^GFP^+^ T cell–derived memory cells, respond rapidly during the early phases of challenge infection. Splenic Ag-experienced CD4^+^YFP^+^GFP^+^ and CD4^+^YFP^+^GFP^−^ T cell subsets were sort purified from *P.yoelii* NL–infected (day 7: 1 × 10^4^ pRBCs [i.v.]) dual reporter mice (CD45.2^+^) and were adoptively transferred, separately, into infected (day 7 postinfection) CD45.1^+^ mice. Recipient mice were treated with pyrimethamine from day 9 of infection for 10 d. Recipient mice were reinfected (1 × 10^4^ pRBCs [i.v.]) on day 60 postinfection with homologous *P. yoelii* NL parasites. (**A**) Total numbers of Ag-experienced CD4^+^YFP^+^GFP^+^ and CD4^+^YFP^+^GFP^−^ T cell–derived memory cells in the spleen and liver of recipient mice on day 60 after primary infection and day 4 of secondary infection. (**B**) Representative histograms and (**C**) frequencies of Ag-experienced CD4^+^YFP^+^GFP^+^ and CD4^+^YFP^+^GFP^−^ T cell–derived memory cells expressing GFP in the spleen and liver of recipient mice on day 60 after primary infection and day 4 of secondary infection. (**D**) Numbers of CD4^+^YFP^+^GFP^+^ and CD4^+^YFP^+^GFP^−^ T cell–derived memory cells expressing YFP (left column) and/or GFP (right column) in the (top row) spleen and (bottom row) liver of recipient mice on day 60 after primary infection and day 4 of secondary infection. (**E** and **F**) Splenic CD4^+^CD11a^+^CD49d^+^ cells were sort purified from mice on day 60 postinfection and total CD4^+^ T cells were purified from uninfected mice (uninf). Cells were stimulated for (E) 24 or 72 h with anti-CD3 and anti-CD28 or (F) 4 h with PMA and ionomycin and supernatant was assayed for (E) IFN-γ production and IL-10 production or (F) IL-2 production. The results are the mean ± SEM of the group with three to five mice per group and are representative of three independent experiments. *p < 0.05. Significance was tested using an unpaired *t* test.

The extensive contraction of the CD4^+^YFP^+^GFP^+^ T cell population, as well as their exhausted phenotype, after malaria infection indicated that the subset may not play a major role in shaping anamnestic immune responses during temporally separated rechallenge infections. Consistent with this scenario, following adoptive transfer (utilizing the same methodology as in Fig. 2), the small number of maintained CD4^+^YFP^+^GFP^+^ T cell–derived memory cells were unresponsive during early secondary infection, failing to proliferate and increase in number in any examined organ (Fig. 4A). Moreover, the CD4^+^YFP^+^GFP^+^ T cell–derived cells also failed to re-express IL-10–GFP following pathogen re-exposure in either the spleen or liver (Fig. 4B–D), with the latter being a major tissue site where IL-10 limits immune-mediated pathology during malaria infection (3–7). These data suggest that CD4^+^YFP^+^GFP^+^ T cell–derived cells are unable to expand and accumulate in number and are not imprinted to immediately replicate their early effector phenotype upon reactivation during reinfection.

In contrast, CD4^+^YFP^+^GFP^−^ T cell–derived memory cells responded rapidly during secondary infection, expanding to a significantly greater extent than CD4^+^YFP^+^GFP^+^ T cell–derived memory cells in the spleen (fold change expansion of 2.3 versus 0.8) and liver (fold change expansion of 5.8 versus 1.0) by day 4 of infection (Fig. 4A). Moreover, CD4^+^YFP^+^GFP^−^ T cell–derived memory cells rapidly upregulated IL-10–GFP expression in the spleen during challenge infection (Fig. 4B, 4C), leading to a significant increase in generation of new CD4^+^YFP^+^GFP^+^ T cells in the spleen during secondary infection (Fig. 4D). Although CD4^+^YFP^+^GFP^−^ T cell–derived memory cells did not upregulate IL-10 expression within the liver during secondary infection (Fig. 4B, 4C), the large expansion of CD4^+^YFP^+^GFP^−^ T cell–derived memory cells within the organ during challenge infection (Fig. 4A) led to a significant increase in the number of CD4^+^YFP^+^GFP^+^ T cells within the liver during secondary infection (Fig. 4D).

Consistent with the reporter data, splenic Ag-experienced CD4^+^ T cells purified on day 60 postinfection (which were predominantly YFP^+^GFP^−^, as shown in Fig. 1C–F) rapidly produced IL-10 and IFN-γ protein following reactivation, producing significantly more of each cytokine than did CD4^+^ T cells purified from naive mice after 24 h anti-CD3 and anti-CD28 stimulation (Fig. 4E). Moreover, the Ag-experienced memory CD4^+^ T cells continued to produce more IL-10 than did naive CD4^+^ T cells following 72 h restimulation, vastly upregulating production from that observed at 24 h (Fig. 4E). In contrast, the memory Ag-experienced CD4^+^ T cells produced equivalent quantities of IFN-γ as did naive CD4^+^ T cells after 72 h restimulation (Fig. 4E). Thus, reactivating malaria infection–induced Ag-experienced memory CD4^+^ T cells appear specifically programmed to rapidly become differentiated IL-10–producing cells following challenge infection. Notably, splenic Ag-experienced CD4^+^ T cells purified on day 60 postinfection also produced large amounts of IL-2 very rapidly upon PMA/ionomycin restimulation (4 h) (Fig. 4F), most likely facilitating their rapid expansion during the early phases of secondary infection, as shown specifically for the CD4^+^YFP^+^GFP^−^ subset (Fig. 4A).

Collectively, these data indicate that CD4^+^YFP^+^GFP^+^ T cell–derived memory cells are unlikely to play a major role in influencing the nature of amnestic immune responses during malaria infection. Instead, CD4^+^YFP^+^GFP^−^ T cell–derived memory cells, due to their rapid ability to produce IFN-γ, IL-2, and proliferate and their capacity to secrete large amounts of IL-10, may play an important role in coordinating inflammation and regulation during the early phases of secondary malaria infections.

### The IL-10 response is quantitatively enhanced in anamnestic immune responses during secondary malaria infection

**FIGURE 5.**
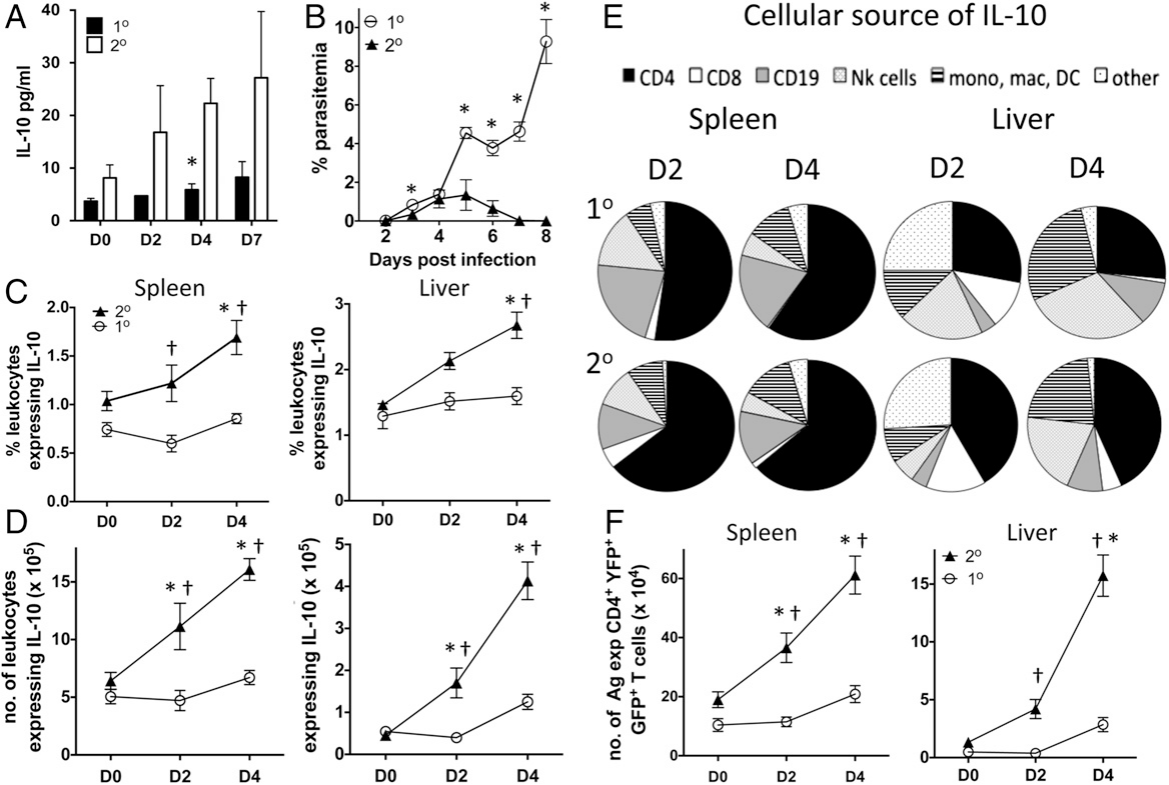
Primary malaria infection leads to reprogramming of the IL-10 response during secondary infection. IFN-γ–YFP and IL-10–GFP dual reporter mice were infected (i.v.) with 1 × 10^4^ *P. yoelii* NL pRBCs or PBS. Mice were treated with pyrimethamine from day 9 postinjection for 10 d before being (re)infected (1 × 10^4^ pRBCs [i.v.]) on day 60 postinfection with homologous *P. yoelii* NL parasites. (**A**) The levels of IL-10 in the plasma of primary- and secondary-infected mice. (**B**) Peripheral parasite levels in primary- and secondary-infected mice. (**C** and **D**) The (C) frequencies and (D) numbers of leukocytes expressing IL-10–GFP in the spleen and liver of primary- and secondary-infected mice. (**E**) The cellular composition of the splenic and hepatic leukocyte IL-10 response in primary- and secondary-infected mice. (**F**) The numbers of Ag-experienced CD4^+^YFP^+^GFP^+^ T cells in the spleen and livers of primary- and secondary-infected mice. The results are the mean ± SEM of the group with three to five mice per group. The results are representative of four independent experiments. *p < 0.05 between day 0 secondary infection versus day 2 or day 4 secondary infection, ^†^*p* < 0.05 between primary infection versus secondary infection on each given day. Significance was tested using a two-way ANOVA test with a Bonferroni post hoc analysis.

Given the ability of memory Ag-experienced CD4^+^ T cells to produce IL-10 during challenge infection, we hypothesized that the dynamics of IL-10 production may be altered in secondary malaria infection compared with primary infection, providing a mechanism underlying infection-induced resistance to severe malarial disease. In agreement with this, circulating plasma IL-10 levels trended higher on days 2 and 7 and were significantly increased on day 4 in secondary-infected dual IFN-γ and IL-10 reporter mice compared with primary-infected counterparts (Fig. 5A). Importantly, peripheral parasitemia was comparable during secondary and primary infections during the early phase of infection (up to and including day 4), after which time secondary-infected mice rapidly cleared peripheral parasites (Fig. 5B). Thus, enhanced expression of IL-10 occurs during the early stages of secondary infection and is not simply a consequence of altered parasite burdens.

Consistent with the plasma IL-10 data, significantly increased frequencies and numbers of total IL-10–GFP^+^ cells were observed in the spleen and liver during the period of patent parasitemia on day 2 and/or day 4 after secondary-infected mice compared with primary-infected mice (Fig. 5C, 5D). Concordant with the upregulation of GFP by CD4^+^YFP^+^GFP^−^ T cell–derived memory CD4^+^ T cells during challenge infection (Fig. 4B), CD4^+^ T cells were the predominant source of IL-10 in both the spleen and liver on days 2 and 4 of secondary infection (Fig. 5E). All GFP-expressing CD4^+^ T cells in the spleen and liver during secondary infection coexpressed YFP, showing that IFN-γ^+^CD4^+^ T cells were also the dominant source of IL-10 during secondary infection (Supplemental Fig. 1). Although CD4^+^YFP^+^ T cells were also the major source of IL-10 in the spleen during the early stages of primary infection, the numbers of splenic CD4^+^YFP^+^GFP^+^ T cells were significantly lower during primary infection than during secondary infection (Fig. 5F). Notably, CD4^+^ T cells constituted a significantly higher fraction of the IL-10^+^ cells in the liver during the early stages of secondary compared with primary infection (Fig. 5E). These data show that IL-10 production, principally from CD4^+^YFP^+^ T cells (most probably reactivated memory cells), is amplified during the early phases of secondary infection compared with primary malaria infection.

### The relative functions of IL-10 during primary and secondary malaria infections

**FIGURE 6.**
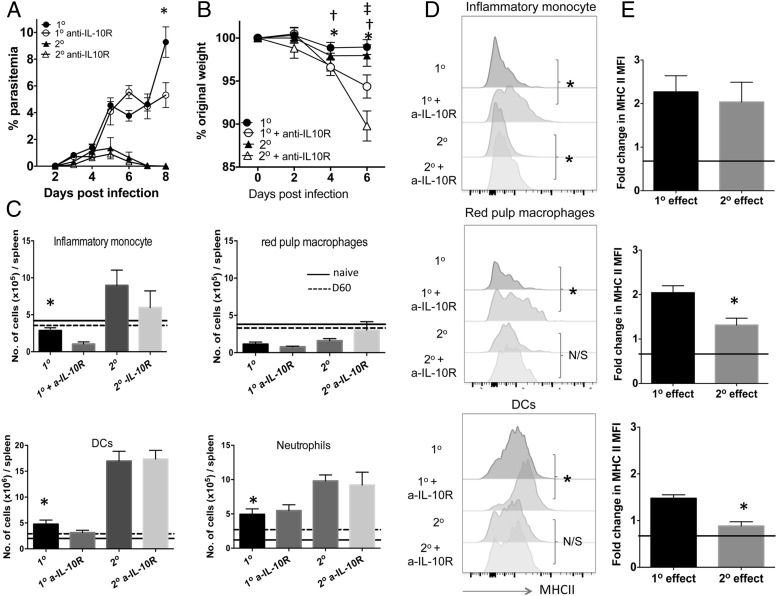
IL-10 exerts strong regulatory effects on the innate compartment during the early phases of primary infection. B6 mice were infected (i.v.) with 1 × 10^4^ *P. yoelii* NL pRBCs or PBS. Mice were treated with pyrimethamine from day 9 postinfection for 10 d before being (re)infected (1 × 10^4^ pRBCs [i.v.]) on day 60 postinfection with homologous *P. yoelii* NL parasites. Mice were injected with 250 μg of anti–IL-10R or PBS 1 d prior to (re)infection and on days 1, 3, and 5 after (re)infection. (**A** and **B**) The course of infection in anti–IL-10R-treated and control primary- and secondary-infected mice was monitored by assessing (A) peripheral parasite levels and (B) weight loss. (**C**) The effect of anti–IL-10R treatment on the numbers of splenic immune cell populations on day 4 during primary and secondary infections. (**D**) Representative histograms showing the effect of anti–IL-10R treatment on MHC II expression (mean fluorescence intensity [MFI]) by splenic innate cells on day 4 during primary and secondary infections. (**E**) Calculated relative effect of anti–IL-10R treatment on MHC II expression by splenic innate cells during primary and secondary infection (presented as fold change in MFI expression compared with expression in respective control-treated primary or secondary-infected mice). Level of no effect is reflected by solid line. (A, C, and E) The results are the mean ± SEM of the group with three to five mice per group and are representative of three independent experiments. (A) *p < 0.05 between primary infection versus anti– IL-10R-treated primary infection on annotated day. (B) *p < 0.05 between day 0 anti– IL-10R-treated primary infection versus anti–IL-10R-treated primary infection on indicated day, ^†^*p* < 0.05 between day 0 anti–IL-10R-treated secondary infection versus anti–IL-10R-treated secondary infection on indicated day, ^‡^*p* < 0.05 between secondary infection versus anti–IL-10R-treated secondary infection on specified day. (A and B) Using two-way ANOVA test with a Bonferroni post hoc analysis. (C) *p < 0.05 between primary infected versus secondary infected, using one-way ANOVA test with a Bonferroni post hoc analysis. (E) *p < 0.05 between fold change in effect during primary versus during secondary infection. (D and E) Using a two-tailed unpaired *t* test.

The altered dynamics of IL-10 production during secondary infection compared with primary malaria infection suggested that IL-10 may exert different quantitative and/or temporal roles during the early phases of primary and anamnestic immune responses to malaria. To assess whether this hypothesis was correct, we administered anti–IL-10R Ab to mice prior to primary or secondary infection and examined the effect on parasite control and the innate and adaptive immune responses. IL-10R blockade did not significantly modify peripheral parasitemia during the early phases of either primary or secondary infection, with an effect observed only during primary infection on day 8 postinfection (Fig. 6A). This was consistent with the unaltered parasite burden observed in IL-10^−/−^ mice compared with WT mice during the early phase of primary *Plasmodium berghei* NK65 infection (35). Ablation of IL-10R signaling did, however, cause significantly increased levels of weight loss during both primary and secondary infection, which was particularly evident during secondary infection on day 6 postinfection (Fig. 6B).

The numbers of inflammatory monocytes (CD68^+^, CD11b^+^, Ly6C^high^), dendritic cells (which include monocyte-derived dendritic cells [CD11c^+^, CD11b^+^, F4-80^−^], classical dendritic cells [CD68^−^, CD11c^+^], and plasmacytoid dendritic cells [CD19^+^, CD11c^+^]), and neutrophils (Ly6G^+^, Ly6C^low^, CD11b^+^, CD68^−^) were significantly increased in spleens of secondary-infected mice compared with primary-infected mice (Fig. 6C), indicating differences in the general nature of the innate immune response during primary and repeat malaria infections. Whereas anti–IL-10R mAb treatment failed to significantly increase the numbers of any of the examined splenic innate populations during primary or secondary infection, including red pulp macrophages (the numbers of which were comparable in primary- and secondary-infected mice [Fig. 6C]), anti–IL-10R significantly elevated the expression of MHC II by inflammatory monocytes during both primary and secondary infections, and by red pulp macrophages and dendritic cells specifically during primary infection (Fig. 6D). Consequently, irrespective of the higher production during secondary infection, IL-10 appears to play a broader and quantitatively stronger role in inhibiting MHC II expression and potentially suppressing the Ag-presenting function of APC populations during the early stages of primary infection compared with secondary malaria infections (Fig. 6E).

**FIGURE 7.**
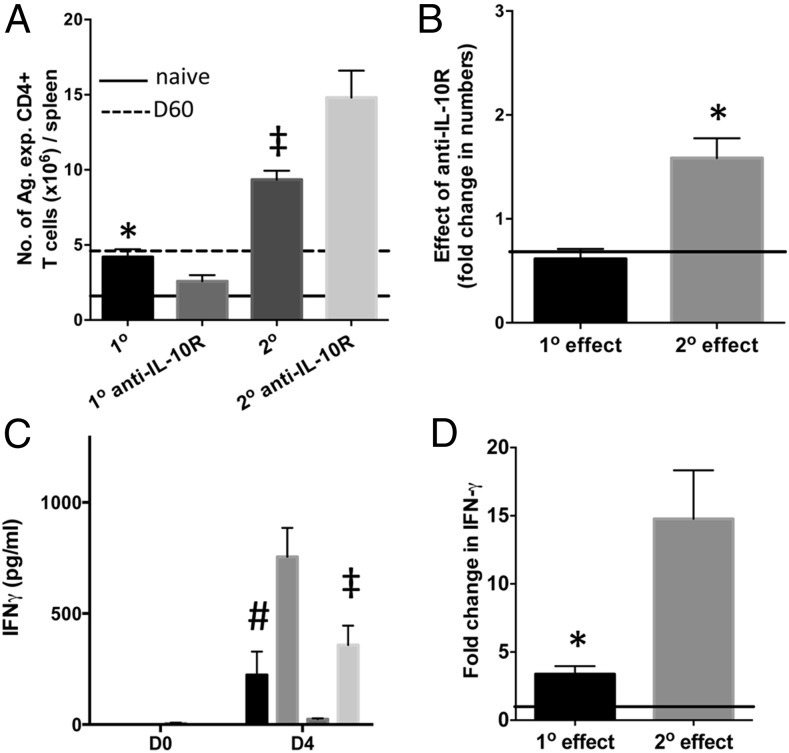
IL-10 exerts strong effects on the adaptive CD4^+^ T cell response during the early phases of secondary malaria infection. B6 mice were injected (i.v.) with 1 × 10^4^ *P. yoelii* NL pRBCs or PBS. Mice were treated with pyrimethamine from day 9 postinjection for 10 d before being (re)infected (1 × 10^4^ pRBCs [i.v.]) on day 60 postinfection with homologous *P. yoelii* NL parasites. Mice were injected with 250 μg of anti–IL-10R or PBS 1 d prior to (re)infection and on days 1, 3, and 5 after (re)infection. (**A**) The effect of anti–IL-10R treatment on Ag-experienced CD4^+^ T cell numbers on day 4 during primary and secondary infections. (**B**) Calculated relative effect of anti–IL-10R treatment on Ag-experienced CD4^+^ T cell numbers during primary and secondary infections (**C**) The effect of anti–IL-10R treatment on the concentration of IFN-γ in the plasma of primary- and secondary-infected mice on day 4 of infection. (**D**) Calculated relative effect of anti–IL-10R treatment on plasma IFN-γ levels on day 4 during primary and secondary infections. (B and D) Presented as fold change in levels in anti–IL-10R Ab-treated mice compared with results in respective control-treated primary- or secondary-infected mice). Level of no effect is reflected by solid line. The results are the mean ± SEM of the group with three to five mice per group and are representative of three independent experiments. (A and C) *p < 0.05 between primary-infected versus secondary-infected mice, ^#^*p* < 0.05 between primary-infected versus anti–IL-10R-treated primary-infected mice, ^‡^*p* < 0.05 between secondary-infected versus anti–IL-10R-treated secondary-infected mice, using one-way ANOVA with a Bonferroni post hoc analysis. (B and D) *p < 0.05 between fold change in effect during primary infection versus during secondary infections, using unpaired *t* test.

Despite the effects on the innate immune response during primary infection, anti–IL-10R administration caused a slight decrease (rather than the expected increase) in the numbers of Ag-experienced CD4^+^ T cells during the early phases of primary infection (Fig. 7A). In contrast, anti–IL-10R administration led to a significant increase in Ag-experienced splenic CD4^+^ T cell numbers during secondary infection (Fig. 7A). Consequently, the effect of anti–IL-10R administration on splenic Ag-experienced CD4^+^ T cell numbers was quantitatively higher during secondary infection compared with primary infection (Fig. 7B). Of note, the numbers of Ag-experienced splenic CD4^+^ T cell cells were significantly increased in secondary-infected mice compared with primary-infected mice, most probably reflecting reactivation of memory CD4^+^ T cells (Fig. 7A). Anti–IL-10R administration did not, however, affect splenic Ag-experienced CD4^+^ T cell activation (as determined by CD69 or ICOS expression) during the early phase of primary or secondary infections, and the activation of splenic Ag-experienced CD4^+^ T cells was not significantly different during primary and secondary infections (results not shown). Consistent with a specific effect of anti–IL-10R administration on the adaptive compartment during secondary infection, anti–IL-10R administration caused a quantitatively larger increase in plasma IFN-γ levels during secondary infection compared with primary infection (Fig. 7C, 7D).

Collectively, these data suggest that IL-10 may have diversified functions within the innate and adaptive immune compartments during primary and secondary malaria infections, respectively. This indicates that different targeted regulatory immune responses may be necessary to modify innate immunity compared with reactivation of memory immune responses during primary and secondary malaria infections, respectively, to prevent formation of infection-induced immunopathology.

## Discussion

IL-10–producing Th1 cells play critical and nonredundant regulatory roles in limiting inflammation and controlling the outcome of many different infections (1, 2, 24, 25). As a consequence, the molecular signals instructing their development have been extensively studied, demonstrating the involvement of myriad cytokines and suites of transcription factors working in coordinated or context-specific pathways (1, 24, 25). However, the stability and memory potential of IL-10–producing Th1 cells after primary infection, as well as their relative influence on the course of secondary infections, have, until now, been significantly understudied.

In this study, we have shown that CD4^+^IFN-γ–YFP^+^IL-10–GFP^+^ T cells have limited capacity to become long-lived memory cells following the resolution of primary malaria infection. Endogenous CD4^+^YFP^+^GFP^+^ T cells were disproportionately lost from the memory-fated and maintained Ag-experienced CD4^+^ T cell population postinfection, as compared with CD4^+^YFP^+^GFP^−^ T cells. Moreover, significantly fewer infection-induced CD4^+^YFP^+^GFP^+^ T cell–derived cells were obtained following adoptive transfer into malaria-infected and drug-cured recipient mice in comparison with total Ag-experienced CD4^+^ T cells or CD4^+^YFP^+^GFP^−^ counterpart cells. Consistently, the total Ag-experienced CD4^+^ T cell population maintained the capacity to produce IFN-γ but rapidly stopped producing IL-10 postinfection.

To our knowledge, we have provided the first evidence that the ephemeral nature of CD4^+^YFP^+^GFP^+^ T cells may be intrinsically dictated by their skewed effector differentiation state, as well as by their apparent cellular exhaustion. The short-lived effector phenotype of CD4^+^YFP^+^GFP^+^ T cells, in comparison with CD4^+^YFP^+^GFP^−^ T cells, is consistent with the idea that IL-10 is expressed only by mature effector T cells following strong TCR activation (24, 25), and that strong T cell activation restricts memory potential (26, 27). Additionally, high expression of markers of exhaustion, including LAG-3, PD-1, and TIGIT, by CD4^+^YFP^+^GFP^+^ T cells potentially restricted their capacity to receive and/or respond to homeostatic signals such as IL-7 during the memory maintenance phase postinfection (36). Of relevance, however, IL-10–producing CD4^+^ T cells have also been shown to express high levels of BLIMP-1 and the aryl hydrocarbon receptor, both of which instruct IL-10 expression in CD4^+^ T cells via direct binding within the gene locus (37, 38). BLIMP-1 and aryl hydrocarbon receptor expression have been shown to antagonize CD4^+^ T cell memory potential in nonmalarial models (39, 40). Thus, the molecular network operational in IL-10–producing CD4^+^ T cells, coupled with their differentiation as short-lived effector cells and the coordinated expression of multiple inhibitory cell surface receptors, may directly restrict their memory potential postinfection. Consequently, it is probable that IL-10–producing CD4^+^ T cells may only be effectively maintained in the presence of malaria Ag in context of TCR engagement. This is consistent with recent data obtained in the field showing that the maintenance of IL-10–producing CD4^+^ T cells is inversely correlated with the duration from previous *P. falciparum* infection (14, 23). Whether IL-10–producing CD4^+^IFN-γ^+^ T cells play an important role in shaping the nature of the memory compartment postinfection, such as has been proposed for Foxp3^+^ regulatory T cells (13, 41), will require further investigation.

Although the restricted life span of YFP^+^GFP^+^ T cells appeared to be a dominant factor driving their loss from the Ag-experienced memory CD4^+^ T cell pool, it was also clear that a high proportion of primary infection–induced CD4^+^YFP^+^GFP^+^ T cells downregulated IL-10–GFP expression following drug-induced resolution of infection. Consequently, a logical conclusion from our results is that IL-10 production by CD4^+^YFP^+^ T cells is inherently unstable during/after malaria infection and that most CD4^+^YFP^+^ T cells do not necessarily become programmed for extended production. Indeed, it would be unexpected for Ag-experienced, including parasite-specific, memory CD4^+^ T cells to continue to produce IL-10, or any nonhomeostatic cytokine, in the absence of inflammation and TCR signals after infection (26, 27, 42). Nevertheless, as the Vert-X mice are transcriptional reporters, our data importantly show that CD4^+^YFP^+^GFP^+^ T cells do not uniformly maintain a poised signature postinfection, where the IL-10 gene is continually transcribed but not actively translated. In agreement with this, it has been suggested that the IL-10 gene does not undergo extensive epigenetic remodeling in memory IFN-γ^+^CD4^+^ T cells (43) but depends upon constant stimulation to maintain IL-10 transcription (44). This is opposed to the archetypal CD4^+^ T cell lineage–defining cytokines IFN-γ or IL-4, which display permissive hypermethylation (H3K4me3) marks in memory cells with Th1 and Th2 ontogenies, respectively (45). Consequently, following transcription in T cells and NK cells, epigenetic remodeling of the IFN-γ gene leads to maintained gene transcription, even though translation may be prevented by destabilizing elements within the mRNA untranslated regions (46). This is consistent with our observation that memory Ag-experienced CD4^+^ T cells from infected/drug-cured mice (most of which are YFP^+^GFP^−^) have the maintained capacity to produce IFN-γ protein immediately ex vivo following mitogenic restimulation. Of note, we have previously shown that cell-sorted IFN-γ–YFP^+^ and IL-10–GFP^+^ T cells actively translate the respective cytokines on day 7 of infection (33). This demonstrates, in agreement with previous studies, that functionally defined mature Th1 cells can be maintained long-term after malaria infection (21).

Despite the limited maintenance of CD4^+^YFP^+^GFP^+^ T cell after malaria infection, we found that the IL-10 response was amplified during secondary malaria infection compared with primary infection. Notably, CD4^+^YFP^+^ T cells were the major source of IL-10 during secondary infection in all examined organs, implying that memory CD4^+^ T cells rapidly expanded and expressed IL-10 during secondary infection. In agreement with this, we found that CD4^+^YFP^+^GFP^−^ T cell–derived memory cells reactivated quickly during secondary infection, proliferating and upregulating IL-10 GFP expression. Of note, the small frequency of GFP^+^ cells within the CD4^+^YFP^+^GFP^−^ T cell–derived memory cell population on day 60 postinfection likely reflects induction of IL-10 in the transferred population prior to drug treatment of the recipient mice (Fig. 4B, 4C). In contrast, consistent with their exhausted phenotype, the small numbers of residual primary infection–derived CD4^+^YFP^+^GFP^+^ T cells were relatively unresponsive during secondary infection. These results suggest that infection-induced CD4^+^YFP^+^GFP^+^ T cells may not play a major role in modulating the course of anamnestic responses during subsequent temporally separated malaria infections.

Importantly, we observed that purified memory Ag-experienced CD4^+^ T cells (most of which were YFP^+^GFP^−^) produced significant quantities of IL-10 protein in vitro following 24 and 72 h restimulation in vitro. This indicates that memory Ag-experienced CD4^+^YFP^+^GFP^−^ T cells are predisposed and programmed toward rapid induction of IL-10 following reactivation during secondary infection. Thus, the enhanced IL-10 response during secondary infection is unlikely to be dominantly or solely controlled by the specific environment the CD4^+^ T cells are exposed to during rechallenge.

Also importantly, the accelerated and amplified IL-10 production during rechallenge did not cause generalized immune inhibition during infection, as innate and adaptive responses were increased during the early phases of secondary infection compared with primary infection. These data are, therefore, inconsistent with a model where generalized suppression of inflammation contributes toward infection-induced resistance to symptomatic malaria. Rather, our data suggest that during secondary malaria infections strong antiparasitic immune responses are allowed to develop to facilitate rapid control and elimination of the parasite, but counterbalancing regulatory responses, of which IL-10 plays a pivotal role, are instructed to ensure that inflammation is restricted below tissue-damaging levels. Such a model is in agreement with recent results showing that upon re-exposure to *P. falciparum*, children develop co-ordinately enhanced antiparasitic and immunoregulatory responses that facilitate parasite control while limiting pathogenic inflammation (23). Of relevance, high ratios of plasma proinflammatory to anti-inflammatory cytokines, including IL-10, are a common feature of severe malaria disease, emphasizing the importance of infection-instructed immune balance in enabling optimal control of malaria infection (9).

Interestingly, our results also highlight distinct activities for IL-10 during primary and subsequent malaria infections. The main function of IL-10 during primary infection appears to be inhibition of MHC II expression by APCs and innate cells, whereas during secondary infection its primarily role appears to be controlling T cell responses and, putatively, the early reactivation of memory CD4^+^ T cells. IL-10 has been previously shown to target and regulate different immune cell populations, depending on the nature of inflammation (47–49), Thus, the varied activity of IL-10 during primary and secondary infections is likely determined by the different immune environments and contrasting responsiveness of immune cells within primary- and secondary-infected hosts. Although the differential sensitivity of recently primed effector T cells and memory CD4^+^ T cells to IL-10 regulation has yet to be examined in detail in any model, IL-10 has been shown to suppress IFN-γ production by TCR-activated memory CD4^+^ T cells (50).

In summary, in this study we have directly shown that malaria infection–induced IL-10–expressing CD4^+^IFN-γ^+^ T cells have limited memory cell potential and do not respond strongly during pathogen re-exposure. These results substantially increase our understanding of the fate of IL-10–expressing CD4^+^ T cells after malaria, and infection in general. In contrast, memory CD4^+^IFN-γ^+^ T cells rapidly expand and produce IL-10 during secondary malaria infection, leading to augmented IL-10 production and coordinated strong antiparasitic and regulatory immune responses. These data provide new insights into the importance and role of IL-10 during primary and repeat malaria infections and reveal the memory CD4^+^ T cell subset that dominantly produces IL-10 to influence the nature of anamnestic immune responses during secondary malaria infection.

## Supplementary Material

Data Supplement
