# Supplementary material for: Long-Lived CD4+IFN-γ+ T Cells rather than Short-Lived CD4+IFN-γ+IL-10+ T Cells Initiate Rapid IL-10 Production To Suppress Anamnestic T Cell Responses during Secondary Malaria Infection
Source: J Immunol. 2016 Sep 14;197(8):3152–64. doi: 10.4049/jimmunol.1600968 (PMC5055201; doi:10.4049/jimmunol.1600968)
Supplement: Data Supplement [file JI_1600968.zip › JI_1600968_Supplemental_Figure_1.pdf]

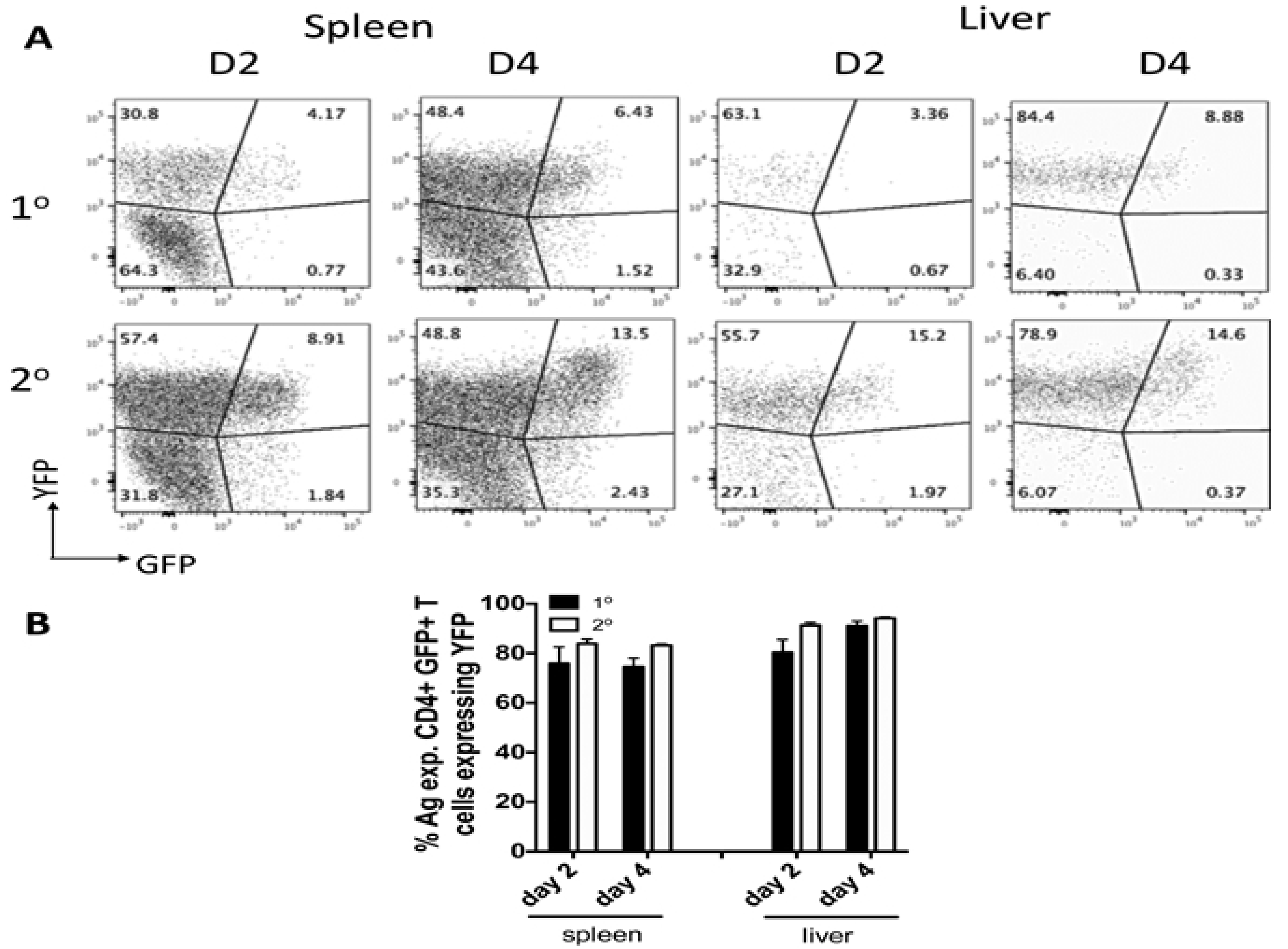

S1 Fig. Antigen-experienced CD4$^+$IFN-γ-YFP$^+$ T cells are the dominant source of IL-10 in the spleen and liver during primary and secondary malaria infection

IFN-γ-YFP and IL-10-GFP dual reporter mice were infected (i.v.) with 1 x10^4 *P. yoelii* NL pRBCs. Mice were treated with Pyrimethamine from day 9 post-injection for 10 days before being (re)infected (1 x10^4 pRBCs [i.v.]) on day 60 post-infection with homologous *P. yoelii* NL parasites. (A) Representative plots showing the expression of YFP and GFP by antigen-experienced (CD11a$^+$CD49d$^+$) CD4$^+$ T cells in the spleen and liver on days 2 and 4 of primary and secondary infection. (B) The proportion of antigen-experienced CD4$^+$ GFP$^+$ T cells in spleen and liver on days 2 and 4 of primary and secondary infection that co-expressed YFP.
